# Neuromuscular characteristics of eccentric, concentric and isometric contractions of the knee extensors

**DOI:** 10.1007/s00421-024-05626-9

**Published:** 2024-10-05

**Authors:** Cassio V. Ruas, Janet L. Taylor, Christopher Latella, G. Gregory Haff, Kazunori Nosaka

**Affiliations:** 1 School of Medical and Health Sciences, Centre for Human Performance, Edith Cowan University, Joondalup, Australia; 2 Neurophysiology Research Laboratory, School of Medical and Health Sciences, Edith Cowan University, Joondalup, Australia; 3 Brazilian Institute of Neuroscience and Neurotechnology, Institute of Physics Gleb Wataghin, Universidade Estadual de Campinas, R. Sérgio Buarque de Holanda, Campinas, São Paulo 77713083-859 Brazil; 4 Neuroscience Research Australia, Randwick, Australia; 5 Directorate of Psychology and Sport, University of Salford, Salford, Greater Manchester UK

**Keywords:** Maximal voluntary contraction, Evoked muscle twitch, Voluntary activation, Corticospinal excitability, Short interval intracortical inhibition

## Abstract

**Purpose:**

We compared voluntary drive and corticospinal responses during eccentric (ECC), isometric (ISOM) and concentric (CON) muscle contractions to shed light on neurophysiological mechanisms underpinning the lower voluntary drive in a greater force production in ECC than other contractions.

**Methods:**

Sixteen participants (20–33 years) performed ISOM and isokinetic (30°/s) CON and ECC knee extensor contractions (110°–40° knee flexion) in which electromyographic activity (EMG) was recorded from vastus lateralis. Voluntary activation (VA) was measured during ISOM, CON and ECC maximal voluntary contractions (MVCs). Transcranial magnetic stimulation elicited motor-evoked potentials (MEPs) and corticospinal silent periods (CSP) during MVCs and submaximal (30%) contractions, and short-interval intracortical inhibition (SICI) in submaximal contractions.

**Results:**

MVC torque was greater (*P* < 0.01) during ECC (302.6 ± 90.0 Nm) than ISOM (269.8 ± 81.5 Nm) and CON (235.4 ± 78.6 Nm), but VA was lower (*P* < 0.01) for ECC (68.4 ± 14.9%) than ISOM (78.3 ± 13.1%) and CON (80.7 ± 15.4%). In addition, EMG/torque was lower (*P* < 0.02) for ECC (1.9 ± 1.1 μV^.^Nm^−1^) than ISOM (2.2 ± 1.2 μV.Nm^−1^) and CON (2.7 ± 1.6 μV^.^Nm^−1^), CSP was shorter (p < 0.04) for ECC (0.097 ± 0.03 s) than ISOM (0.109 ± 0.02 s) and CON (0.109 ± 0.03 s), and MEP amplitude was lower (*P* < 0.01) for ECC (3.46 ± 1.67 mV) than ISOM (4.21 ± 2.33 mV) and CON (4.01 ± 2.06 mV). Similar results were found for EMG/torque and CSP during 30% contractions, but MEP and SICI showed no differences among contractions (p > 0.05).

**Conclusions:**

The lower voluntary drive indicated by reduced VA during ECC may be partly explained by lower corticospinal excitability, while the shorter CSP may reflect extra muscle spindle excitation of the motoneurons from vastus lateralis muscle lengthening.

**Supplementary Information:**

The online version contains supplementary material available at 10.1007/s00421-024-05626-9.

## Introduction

Muscle force generated during eccentric (lengthening muscle) contractions is generally greater than that of isometric or concentric (shortening muscle) contractions (Nuzzo et al. 2023). For instance, a classic study by Katz (1939) showed that active lengthening force of a frog gastrocnemius was 1.1–1.8 times greater than the force during isometric contraction. Lombardi & Piazzesi (1990) also reported that steady lengthening of isolated frog muscle fibers from a plateau of isometric tetanic force increased tension up to twofold. It has been suggested that the combination of extra cross-bridge attachments (i.e., myosin heads bind and re-bind to actin filaments) and the stiffening of titin (i.e., titin winds upon the actin filament when it is rotated by myosin) contribute to the greater force production during eccentric than concentric and isometric contractions (Franchi et al. 2017; Nishikawa et al. 2012).

In comparison to the animal studies mentioned above, the force generated by humans during maximal voluntary contraction (MVC) does not always differ substantially between eccentric and isometric contractions. For example, Beltman et al. (2004) reported that average ± SD MVC torque of the knee extensors of young men was 270 ± 55 Nm for eccentric contractions and 252 ± 47 Nm for isometric contractions. Similarly, Duchateau & Enoka (2016) highlighted that the peak force achieved by untrained individuals during eccentric MVC is usually either comparable to, or less than 40% greater than that in isometric or slow concentric contraction. Franchi et al. (2017) suggested that these differences might be due to inhibitory mechanisms within the central nervous system during eccentric contractions in humans.

The level of muscle activation is shown to be lower during eccentric contractions when compared with isometric and concentric contractions (Duchateau and Baudry 2014). In MVCs of the knee extensor muscles, electromyographic (EMG) activity was reported as 7–51% lower during eccentric than concentric contractions at angular velocities ranging from 30° to 150°/s (Kellis and Baltzopoulos 1998). Beltman et al. (2004) showed that the voluntary activation (VA) was 2.5–3 times lower during eccentric than concentric or isometric contractions. During both maximal and submaximal eccentric contractions, reductions in EMG amplitude and voluntary drive are likely a result of reduced motor unit recruitment and discharge rate, involving either spinal and/or supraspinal mechanisms that reduce neural drive to the muscle (Duchateau and Baudry 2014; Duchateau and Enoka 2016; Colard et al. 2023; Glories and Duclay 2023; Barrué-Belou et al. 2018, 2019). Other mechanisms include increased presynaptic inhibition of muscle spindle afferents, and/or a reduction of descending drive to the motoneurons (Hahn et al. 2012; Duclay et al. 2014). The latter could result from increased inhibition within the motor cortex and/or reduced corticospinal excitability (Duchateau and Enoka 2016; Duchateau and Baudry 2014).

Findings with regard to cortical activity, excitability and inhibition during eccentric contractions are difficult to interpret. For instance, electroencephalography and functional magnetic resonance imaging show more cortical activity during submaximal eccentric than concentric contractions (Fang et al. 2001, 2004; Yao et al. 2014). In contrast, motor-evoked potentials (MEPs) elicited by transcranial magnetic stimulation (TMS) are often smaller during eccentric than concentric contractions (Abbruzzese et al. 1994; Gruber et al. 2009). The corticospinal silent period (CSP), a period of reduced EMG activity following transcranial stimulation, has been reported as shorter (less inhibition) during eccentric than concentric contractions of the plantar flexors (Duclay et al. 2014). In contrast, the CSP has been shown to be longer (more inhibition) during eccentric than concentric contractions of the first dorsal interosseous muscle (Opie and Semmler 2016). For quadriceps, the CSP has been reported to be shorter for eccentric than concentric contractions assessed in mid-range (75° of knee flexion), but longer when TMS was delivered with the muscle at a long length (100° of knee flexion) (Doguet et al. 2017). They speculated that the greater CSP found at long muscle lengths was a result of a greater reduction in descending transmission to the motoneurons (Doguet et al. 2017). Short interval intracortical inhibition (SICI) assessed with paired pulse TMS during low-intensity first dorsal interosseous muscle contractions showed the least inhibition during concentric contractions, more during eccentric contractions, and the most inhibition during isometric contractions (Opie and Semmler 2016). It appears that the inhibitory behavior depends on the muscle and the measurement conditions. In addition, disparate approaches and stimulation parameters may affect inhibitory responses (Brownstein et al. 2018; Ruas et al. 2020). Thus, it is difficult to make proper comparisons between studies because of different methodologies.

Nevertheless, the knee extensor muscles appear to present different somatotopic organization, functional roles and recruitment thresholds, which may result in distinct inhibitory behavior when compared to upper limb muscles (Ruas et al. 2020; Krishnan 2019; Leung et al. 2018). Although lower voluntary drive has been previously reported for the knee extensors (Kellis and Baltzopoulos 1998; Beltman et al. 2004), it is not clear whether less corticospinal excitability or increased intracortical inhibition contribute to the reduced descending drive and poor voluntary activation in eccentric contractions of the quadriceps muscles. To clarify these, neurophysiological measures using TMS may shed light on the intracortical and corticospinal behavior during knee extensor eccentric contractions in comparison to isometric and concentric contractions.

Therefore, the present study compared the level of voluntary drive, and intracortical and corticospinal responses to TMS such as MEP, CSP and SICI during eccentric, isometric and concentric knee extensor contractions to explore neuromuscular and neurophysiological mechanisms that underly the lower muscle activation commonly reported in eccentric contractions. Since intracortical inhibition may be down-regulated to preserve corticospinal output to the target muscle during maximal contractions (Hendy et al. 2019; Ortu et al. 2008), we compared SICI between the contraction types during submaximal contractions (30% MVC) only. Furthermore, we examined whether individuals who could produce higher eccentric torque relative to isometric torque had less inhibition within the motor cortex and/or greater corticospinal excitability, leading to increased neural drive to the muscle. Our hypothesis was that the amount of SICI would be greater (i.e., more intracortical inhibition) during eccentric than other contractions of the knee extensor muscles. We aimed to provide novel insights to the literature regarding intracortical and corticospinal mechanisms that contribute to the modulation of motor output during eccentric contractions of the knee extensors.

## Methods

### Participants

A total of 16 (12 male and 4 female) participants who were regularly performing physical or recreational sporting activities were recruited for the study. Their average ± SD (range) age, height and body mass were 26.9 ± 4.0 (20–32) years, 1.73 ± 0.01 (1.52–1.84) m, and 80.4 ± 17.2 (59.2 – 111.7) kg, respectively. The sample size was based on the difference between MVC torque in eccentric (287.7 ± 47.0 Nm), isometric (246.9 ± 50.0 Nm) and concentric contractions (216.7 ± 45.2 Nm) of the knee extensors in young adults from a previous study (Ruas et al. 2018), which provided the Cohen’s effect size of 0.8. Using G*Power 3.1 (Institute for Experimental Psychology, Dusseldorf, Germany), with a power of 0.8 and a significance level of 0.05, it was estimated that at least 12 participants were necessary to identify potential differences in MVC torque among the three contraction types. Accounting for a potential estimation error in the sample size calculation, and possible withdrawal of some participants from the study, 16 participants were recruited.

Participants provided written informed consent and completed a pre-exercise medical questionnaire. To identify leg dominance, the Waterloo Footedness Questionnaire Revised (van Melick et al. 2017) was completed by the participants, and only the dominant leg was assessed in all tests. All participants were identified as right leg dominant. Ethical approval was obtained from the Edith Cowan University Human Research Ethics Committee (Project no. 00944).

### Experimental design

All participants visited the laboratory on one occasion and completed the following assessments in order: (1) MVC torque of the knee extensors of the leg for isometric and isokinetic concentric and eccentric contractions; (2) isometric, concentric and eccentric contractions at 30% of MVC (i.e., submaximal contractions) with superimposed single- and paired-pulse TMS; (3) isometric, concentric and eccentric MVCs with superimposed peripheral electrical nerve stimulation; (4) additional isometric, concentric and eccentric MVCs with superimposed single-pulse TMS. The order of isometric, concentric and eccentric contractions was randomized for each block of the measures shown above and among participants.

Neurophysiological responses were recorded via surface EMG from the vastus lateralis muscle. Outcome measures included MVC torque of eccentric, isometric and concentric contractions, and other neuromuscular indices [i.e., MEP, SICI, CSP duration, resting twitch torque, superimposed twitch torque, VA, EMG activity, Maximal M wave (M_MAX_)] during maximal and/or submaximal contractions which were compared across contraction modes.

### Experimental setup

All participants sat upright on an isokinetic dynamometer (Biodex System 4 Pro, Shirley, NY) for all assessments with the hips positioned at 85° of hip flexion (0° = full hip extension), and had straps across their chest, hips and thighs to minimize movement of other parts of the body. The lateral epicondyle of the femur from the participant’s dominant leg was aligned to the dynamometer’s axis of rotation, with the leg attached to the lever arm 2 cm above the medial malleolus (Doguet et al. 2017).

During all testing, EMG activity from the vastus lateralis muscle was recorded by a PowerLab EMG system with a 16-bit analog-to-digital converter (PowerLab 16/35, ADInstruments, 457 Bella Vista, NSW, Australia) using surface electrodes (Ag–AgCl; Ambu Blue Sensor N-00-S/25, Ambu, Denmark). A LabChart software (ADInstruments, Bella Vista, NSW, Australia) was used to record EMG and torque signals at a sampling rate of 2-kHz (common mode rejection ratio > 85 dB, gain = 1000), which also automatically triggered the transcranial and peripheral nerve stimuli. For all participants, one electrode was placed at ~ 66% of the line between the inguinal crease and the patella and the second, 5 cm distal, in a pseudo-monopolar orientation (Ruas et al. 2022a, b). A ground electrode was placed over the tibial tuberosity of the tested leg, after the areas were shaved, abraded and cleaned to reduce impedance (Z < 5 kΩ).

Raw EMG signals were filtered (20–1000 Hz band pass filter) and amplified (1000x). Root mean square (RMS) EMG was calculated over a 500 ms period prior to stimulation during eccentric, isometric and concentric contractions. RMS was also calculated around the time of peak torque during MVCs without the stimulation to ensure the torque, EMG and knee angle recordings were matched. For the submaximal contractions, target torques of 30% MVC were set for each participant, because the 30% level allowed the participants to maintain the target torque for the entire range of motion (between 110° and 40° of knee flexion) tested. The EMG values prior to simulation were divided by the torque at 30% MVC for each contraction and averaged. For MVCs, the EMG values of the MVCs (without stimulation) that presented the highest peak torque of the three trials of each contraction type tested were selected and divided by the peak torque of that MVC (EMG/torque) (Duclay et al. 2011).

### Maximal voluntary contraction (MVC) torque

Participants performed eccentric, isometric and concentric MVCs unilaterally with the dominant leg (the preferred leg for kicking a ball) in a randomized order on the isokinetic dynamometer. Isometric MVC consisted of three repetitions of 3 s with a 2-min rest between repetitions with the leg positioned at 75° of knee flexion (0° = full knee extension) (Doguet et al. 2017; Brown and Weir 2001). The angle (75°) has been reported to represent an intermediate muscle length of vastus lateralis and is within the optimal range for isometric knee extension torque production (Doguet et al. 2017; Becker and Awiszus 2001; Pietta-Dias et al. 2020).

Eccentric and concentric MVCs were performed at 30°/s through 70° of range of motion (between 110° and 40° of knee flexion) on the isokinetic dynamometer (Doguet et al. 2017). Both eccentric and concentric MVC measures consisted of three trials each with a 3-min rest between trials and conditions. Two practice contractions with a 3-min rest between each were given before the start of each set of muscle contractions to ensure that the maximal effort contractions were performed by each participant. In the eccentric trials, participants were asked to perform an isometric MVC at 40° of knee flexion first (Jensen et al. 1991) for ~ 1 s, and then resist the movement of the machine as hard as possible (Brown and Weir 2001). For the concentric trials, participants were asked to push the lever arm as hard and as fast as possible starting at 110° of knee flexion. The MVC that presented the highest peak torque of the three trials for each contraction mode was used for further analysis. To assess the magnitude of neuromuscular fatigue, the isometric MVC torque was also measured at the end of the exercise session following the same protocol described above. Based upon the highest peak torque, target torques of 30% of MVC were calculated for later assessments, and the ratio of eccentric MVC torque relative to isometric MVC torque (ECC/ISOM) was calculated for later analysis.

### Peripheral nerve stimulation

Peripheral nerve stimuli were delivered over the femoral nerve to evoke M-waves, resting and superimposed twitches. Electrical stimuli were delivered by a constant-current stimulator (DS7AH, Digitimer, Welwyn 369 Garden City, UK) using a cathode and anode (White Sensor 4560 M, 79 mm, Ambu, Ballerup, Denmark) placed over the femoral triangle and greater trochanter, respectively (Ruas et al. 2020). Single, electrical stimuli with a duration of 200 μs and increasing intensity were delivered until a maximal amplitude of the M-wave (M_MAX_) was reached for the vastus lateralis muscle with the knee at rest and passively supported at 75° of flexion (Doguet et al. 2017; Duclay et al. 2011). The superimposed twitch and resting twitch torques were assessed using a supramaximal stimulus intensity (equivalent to 150% of M_MAX_ intensity). This stimulus was automatically delivered at 75° of knee flexion during isometric, and eccentric and concentric MVCs at 30°/s to elicit superimposed twitch, and also at ~ 2 s after each MVC with the muscles relaxed to elicit a potentiated resting twitch. After isometric MVCs, the leg remained at 75° of flexion, whereas after eccentric and concentric MVCs, the dynamometer returned the leg to its starting position and then moved it passively through the 70° range (lengthening or shortening the knee extensors also at 30°/s with stimuli automatically delivered at 75°). Three trials consisting of superimposed twitch and resting twitch stimuli were given for each contraction type. Resting twitch and superimposed twitch torque amplitudes were measured as the difference between the torque just prior to the onset of the twitch (i.e., approximately 12 ms after the femoral nerve stimulation) and the peak torque of the twitch. VA was further determined by calculating (1 – superimposed twitch torque/resting twitch torque) × 100. For each contraction type, three values of each parameter [i.e., resting twitch torque (Nm), superimposed twitch torque (Nm), peak-to-peak amplitude (mV) of M_MAX_, and VA (%)] were averaged and used for further comparisons.

### Transcranial magnetic stimulation (TMS)

TMS (Magstim 200^2^, Magstim Co, Dyfed, UK) was delivered with a 110 mm double cone coil. First, the ‘hotspot’ of the vastus lateralis was found using a stimulation intensity that elicited a small response in the muscle with the knee relaxed and passively supported at 75° of flexion (Doguet et al. 2017). The hotspot was determined as the area that evoked the greatest MEP amplitude with a given stimulation intensity. To determine the hotspot, the coil was placed at the vertex and then moved in medio-lateral and/or anterior–posterior directions by 1-cm steps until finding the greatest stimuli response (Ruas et al. 2020). Then, the active motor threshold was determined as the intensity at which at least 5 of 10 stimuli evoked a MEP (peak-to-peak amplitude > 200 µV) (Rothwell et al. 1999) while individuals performed a knee extensor isometric contraction equal to 10% of isometric MVC at 75° of knee flexion. The TMS intensity was then increased to 140% of active motor threshold (considered as an intensity within the rising phase of stimulus response curve) and kept constant throughout the protocol regardless of contraction type (Doguet et al. 2017; Rossini et al. 2015).

During testing, TMS was delivered during submaximal contractions. Five single- and five paired-pulse TMS were delivered during 10 separate 30% MVCs for each type of muscle contraction (i.e., eccentric, isometric and concentric). Paired-pulse TMS used a subthreshold of 74% of the active motor threshold conditioning pulse and 140% of the active motor threshold test pulse (interstimulus interval of 2 ms) to assess SICI. The conditioning pulse intensity was based on our previous study (Ruas et al. 2020) that found an average conditioning intensity of 74% of the active motor threshold eliciting ~ 50% of maximal inhibition for individuals when measuring SICI related to the vastus lateralis. Contraction torque targets for each individual were set as 30% of the isometric, concentric and eccentric MVCs recorded at the start of the experimental session. For the eccentric and concentric isokinetic contractions, TMS and peripheral nerve stimuli were externally triggered so that they were delivered automatically for each repetition at 75° of knee flexion (Doguet et al. 2017). Single pulse MEP peak-to-peak amplitudes were averaged. Paired pulse MEP amplitudes were averaged and expressed as a percentage of the single pulse MEPs for each contraction mode.

Single pulse TMS was also delivered during isometric MVCs and eccentric and concentric MVCs at 30°/s (five repetitions for each MVC in a block randomized order). Based on the study by Hahn et al. (2012), at least 3 min of rest between MVCs and between contraction modes was provided in order to minimize fatigue. MEP peak-to-peak amplitude during MVCs was averaged for each contraction type.

CSP duration was also calculated from single-pulse TMS delivered during submaximal and maximal contractions as the time interval between the stimulus and the return of EMG activity (i.e., 50% of its background value over 100 ms period prior to stimulation) (Butler et al. 2012). The average CSP duration for each contraction mode tested was calculated.

### Statistical analyses

Data were first screened using a Shapiro–Wilk test, which confirmed that all data were normally distributed. The absolute values of MVC torque, peak torque angle of MVC, MEP, SICI, CSP duration, EMG (RMS), M_MAX_, superimposed twitch torque, resting twitch torque and VA of participants were compared between eccentric, isometric and concentric contractions by one-way repeated measures ANOVA for each variable. Based on the eccentric MVC torque of the knee extensors in relation to the isometric MVC torque, the same dependent variables were compared between two groups of individuals according to their ECC/ISOM MVC torques.

In order to standardize the groups, the ECC/ISOM MVC of the participants was transformed and standardized to Z-scores, resulting in a common group standard mean value = 0, and a standard deviation value = 1. Individuals presenting a z-score smaller than the mean of 0 (i.e., – 0.411 to – 1.689) were considered as individuals with low ECC/ISOM MVC torque (Group A; n = 7; range 90.0 to 109.7%), and those that presented a z-score greater than the mean of 0 (i.e., 0.001 to 2.88) were considered as individuals with high ECC/ISOM MVC torque (Group B; n = 9; range 111.1 to 186.1%). The variables were compared between the two groups by two-way ANOVAs with contraction type as a repeated measures factor. If significant F values were found, results were followed up with least significant difference post-hoc analysis. A Greenhouse–Geisser correction was used if sphericity was violated. Isometric MVC torques at the beginning and end of the session were also compared by a paired t-test to determine if fatigue had occurred. Significance level was set at *p* < 0.05. All analyses were performed with SPSS 21.0 (Statistical Package for Social Sciences, Chicago, IL, USA). Percentage differences among the three contraction modes are also reported in the results.

## Results

### MVC torque

**Fig. 1:**
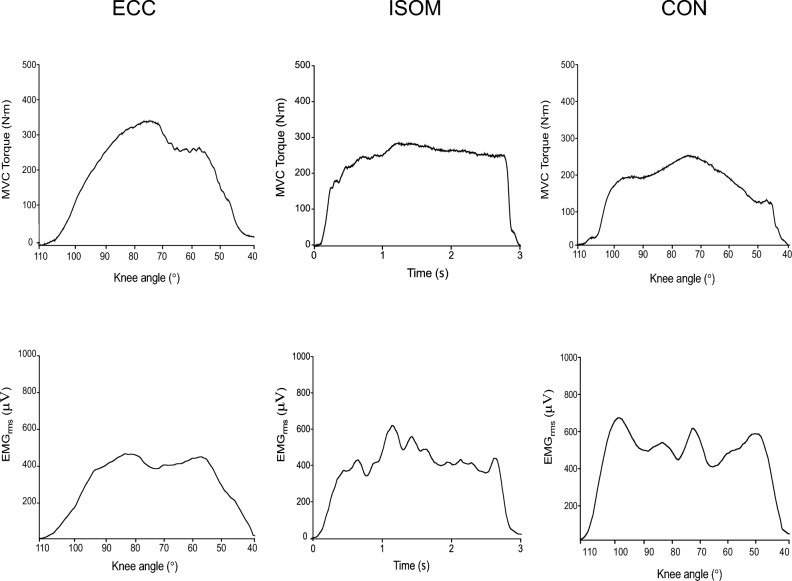
Eccentric (ECC), isometric (ISOM) and concentric (CON) maximal voluntary contraction (MVC) torque and vastus lateralis muscle electromyographic (EMG) activity root mean square (RMS) raw traces of a single participant

**Fig. 2:**
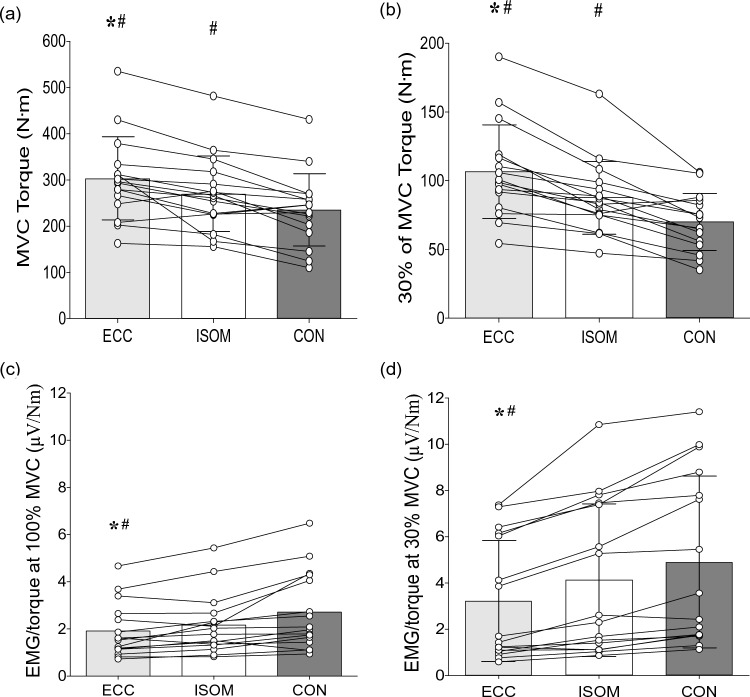
Maximal voluntary contraction (MVC) torque (**a**) and 30% MVC torque (**b**), and vastus lateralis muscle electromyographic (EMG) activity normalized to torque (EMG/torque) at 100% MVC (**c**) and EMG/torque at 30% MVC (**d**) during eccentric (ECC), isometric (ISOM) and concentric (CON) contractions of the knee extensors of individuals. Bars indicate mean ± SD values of 16 participants, and circles represent individuals. * Indicates significant difference from ISOM (P < 0.05). # indicates significant difference from CON (*P* < 0.05)

Eccentric, isometric and concentric MVC raw traces of a single participant whose MVC values were similar to those of the group average are shown in Fig. 1. Significant differences among eccentric (average ± SD: 303 ± 90 Nm, range 163–546 Nm, peak torque angle: 83 ± 9°), concentric (235 ± 79 Nm, 110–431 Nm, 84 ± 9°) and isometric (270 ± 82 Nm, 156–482 Nm) contractions were evident for MVC torque (*F*_2,30_ = 24.40, *η*^2^_p_ = 0.62, *p* < 0.001) without a significant difference between concentric and eccentric peak torque angles (*p* = 0.77). Eccentric MVC torque was 13.8 ± 21.2% (range – 10.0 to 86.1%, *p* < 0.001) greater than isometric MVC torque, and 32.7 ± 27.5% (range – 9.4 to 113.8%, *p* = 0.004) greater than concentric MVC torque (Fig. 2a). Isometric MVC torque was also 17.2 ± 17.0% (range – 8.5 to 46.3%) greater than concentric MVC torque (*p* = 0.001). Similarly, the 30% MVC torque was greater (*F*_2,30_ = 28.75, *η*^2^_p_ = 0.66, *p* < 0.001) during eccentric (104.0 ± 33.6 Nm, 54.3–184.5 Nm) than isometric (87.5 ± 26.6 Nm, 47.3–163.2 Nm, *p* < 0.001) and concentric (73.1 ± 20.3 Nm, 43.0–108.8 Nm, *p* < 0.001) contractions, and greater (*p* = 0.002) for isometric than concentric contractions (Fig. 2b).

No significant difference was found for isometric MVC torque measured at the beginning and the end of the session (*p* = 0.115). This indicated that the number of muscle contractions performed during the session did not cause detectable neuromuscular fatigue and thus, was unlikely to have impacted any of the responses observed.

### EMG activity

Figure 1 shows eccentric, isometric and concentric EMG (RMS) raw traces of a single participant who represented the group average MVC values. Absolute EMG (RMS) amplitudes during eccentric, isometric, and concentric MVCs were 559 ± 329 µV, 584 ± 383 µV, and 607 ± 373 µV, respectively, and no significant difference was evident among contractions (*F*_2,30_ = 1.32, *η*^2^_p_ = 0.081, *p* = 0.283). However, when EMG activity was normalized to torque for each contraction (EMG/torque), significant (*p* < 0.001) differences were evident across eccentric, isometric and concentric contractions at 100% MVC (*F*_2,30_ = 15.96; *η*^2^_p_ = 0.52, Fig. 2c) and 30% MVC (*F*_2,30_ = 24.26, *η*^2^_p_ = 0.62, Fig. 2d). EMG/torque at 100% eccentric MVC was 16.5 ± 23.2% less than isometric MVC, and 43.0 ± 33.4% less than concentric MVC (both *p* < 0.001). EMG/torque at 100% isometric MVC was also 24.6 ± 29.1% less than concentric MVC (*p* = 0.004). Similarly, EMG/torque at 30% eccentric MVC was 27.1 ± 16.3% less than that of isometric, and 63.6 ± 24.8% less than that of concentric contraction (both *p* < 0.001). EMG/torque at 30% isometric MVC was also 30.3 ± 23.0% less than 30% concentric MVC (*p* = 0.001). These differences were shown by most of the participants (Fig. 2d).

### Resting twitch torque, superimposed twitch torque and VA

**Fig. 3:**
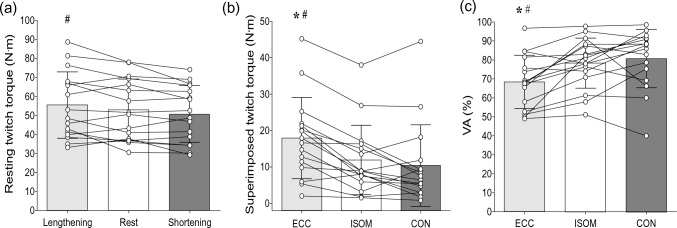
Resting twitch torque (**a**) during passive lengthening, shortening and rest conditions, and superimposed twitch torque (**b**) and voluntary activation (VA, **c**) during eccentric (ECC), isometric (ISOM) and concentric (CON) muscle contractions of the knee extensors of individuals. Bars indicate mean ± SD values of 16 participants, and circles represent individuals. * Indicates significant difference from ISOM (*P* < 0.05). # indicates significant difference from CON or passive shortening (*P* < 0.05)

Significant differences among eccentric, isometric and concentric trials were evident for resting twitch torque (*F*_2,30_ = 5.20, *η*^2^_p_ = 0.26, *p* = 0.011) (Fig. 3a), superimposed twitch torque (*F*_2,30_ = 23.13, *η*^2^_p_ = 0.61, *p* < 0.001) (Fig. 3b) and VA (F_2,30_ = 11.20, *η*^2^_p_ = 0.43, *p* < 0.001) (Fig. 3c). Resting twitch torque was greater (*p* = 0.014) during passive lengthening than shortening by 7.3 ± 12.0%, but no significant difference was found between lengthening and isometric conditions (*p* = 0.122). The superimposed twitch torque was greater (both *p* < 0.001) for eccentric than isometric contractions by 34.0 ± 28.0% and concentric contractions by 48.9 ± 22.2%. VA was lower (both *p* < 0.002) for eccentric than isometric contractions by 16.3 ± 18.1% and concentric contractions by 19.5 ± 22.0%. Based upon the post-hoc analyses, there were no differences (all *p* > 0.622) between concentric (shortening) and isometric contractions for resting twitch torque, superimposed twitch torque, and VA.

### MEP, M_MAX_, SICI, and CSP duration

**Fig. 4:**
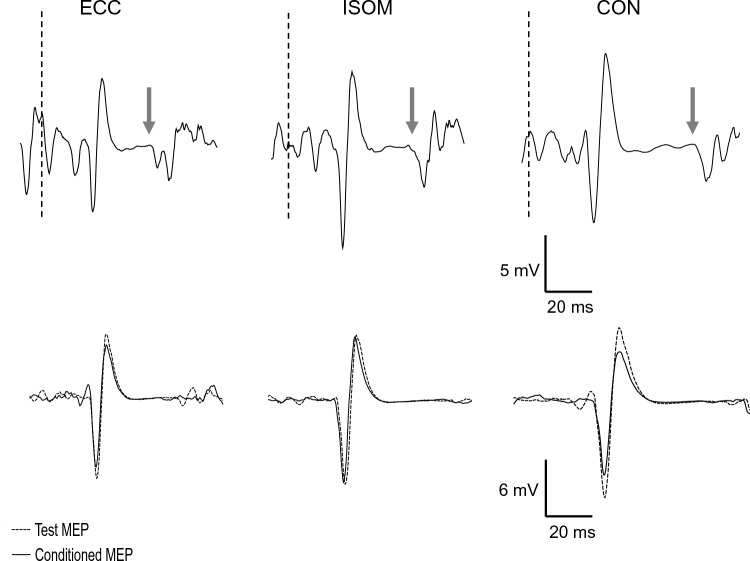
Raw traces of vastus lateralis motor-evoked potentials (MEP) and corticospinal silent periods (CSP) amplitudes during maximal voluntary eccentric (ECC), isometric (ISOM) and concentric (CON) contractions, and short-interval intracortical inhibition (SICI) amplitudes during 30% maximal voluntary eccentric (ECC), isometric (ISOM) and concentric (CON) contractions for a single participant. Dashed lines indicate the time of stimulation, and arrows indicate the end of CSP period following MEPs. SICI traces are shown in pairs for test (dotted lines) and conditioned (solid lines) MEPs

**Fig. 5:**
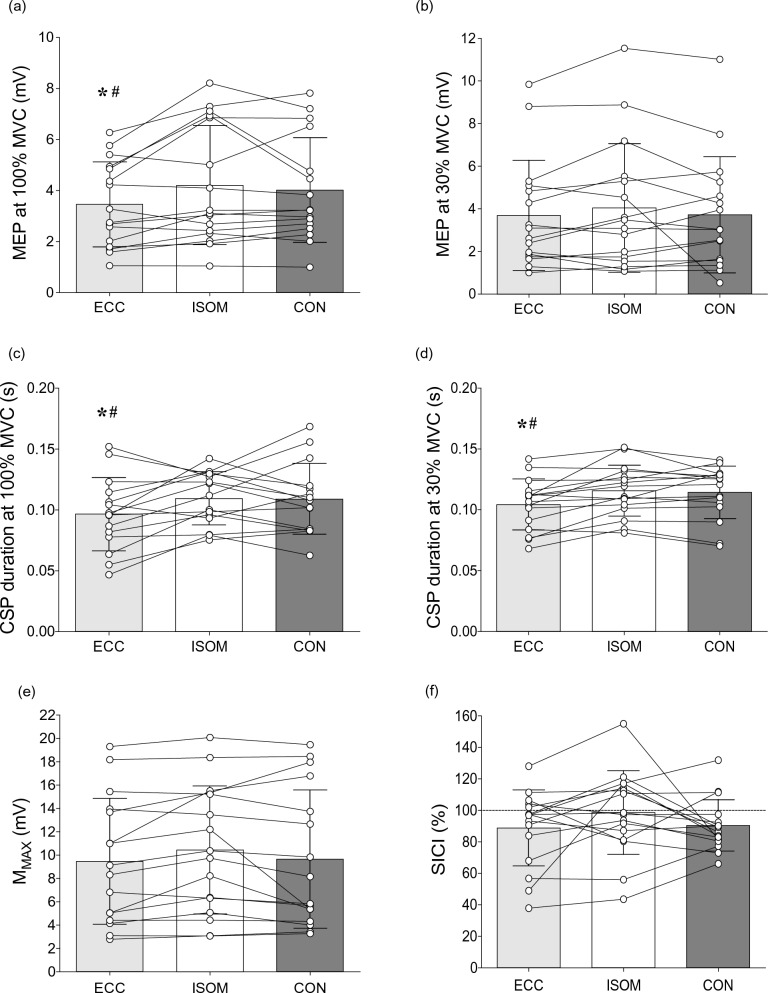
Motor-evoked potential (MEP) peak-to-peak amplitudes at 100% MVC (**a**) and 30% MVC (**b**), corticospinal silent period (CSP) duration at 100% (**c**) and 30% (**d**) of MVC, M_MAX_ (**e**), and short-interval intracortical inhibition (SICI; paired-pulse/single pulse MEP) (**f**) during eccentric (ECC), isometric (ISOM) and concentric (CON) muscle contractions of the knee extensors of individuals. Bars indicate mean ± SD values of 16 participants, and circles represent individuals. * indicates significant difference from ISOM (*P* < 0.05). # indicates significant difference from CON (*P* < 0.05)

MEP and CSP amplitudes during MVCs, and SICI amplitudes during 30% MVC in the three contraction types for a single participant who represents the group are shown in Fig. 4. As shown in Fig. 5, no significant differences between eccentric, isometric and concentric contractions were found for M_MAX_ during MVC (*F*_2,30_ = 2.12, *η*^2^_p_ = 0.12, *p* = 0.138), M_MAX_ at rest (F_2,30_ = 3.04, *η*^2^_p_ = 0.02, *p* = 0.740), and SICI (*F*_2,30_ = 1.29, *η*^2^_p_ = 0.08, *p* = 0.289). Since there were no differences in M_MAX_ at MVC and at rest across contractions, EMG and MEPs (not normalized to M_MAX_) were considered for the statistical analyses. No significant differences between contractions were found for MEP at 30% MVC (*F*_2,30_ = 0.81,, *η*^2^_p_ = 0.05, *p* = 0.453), but significant differences were evident for MEP at 100% MVC (F_2,30_ = 5.32, *η*^2^_p_ = 0.26, *p* = 0.011). MEP amplitude during MVC was lower (both p < 0.015) for eccentric than isometric contractions by 20.1 ± 22.8%, and concentric contractions by 17.5 ± 21.2%.

Significant differences among eccentric, isometric and concentric trials were evident for CSP duration during MVC (*F*_2,28_ = 3.66, *η*^2^_p_ = 0.21, *p* = 0.039) and CSP during 30% MVC (*F*_2,30_ = 9.46, *η*^2^_p_ = 0.39, *p* = 0.001). One participant relaxed immediately after MEPs were evoked during concentric, eccentric and isometric MVCs, so the CSP duration during MVC was only analyzed in 15 participants. CSP duration during MVC was shorter (p < 0.042) for eccentric than isometric contractions by 19.2 ± 25.8% (range – 69.4 to 16.3%), and concentric contractions by 17.0 ± 23.7% (range – 64.1 to 17.9%). Similarly, during 30% contractions, CSP duration was shorter (P < 0.003) for eccentric than isometric contractions by 11.9 ± 12.7% (range – 36.4 to 3.4%) and concentric contractions by 10.1 ± 11.8% (range – 34.9 to 16.0%). Based upon the post-hoc analyses, there were no differences (*p* > 0.624) among isometric and concentric for CSP duration during MVC and 30% contractions (Fig. 5c, d).

### Comparison among individuals based on ECC/ISOM MVC torque

**Fig. 6:**
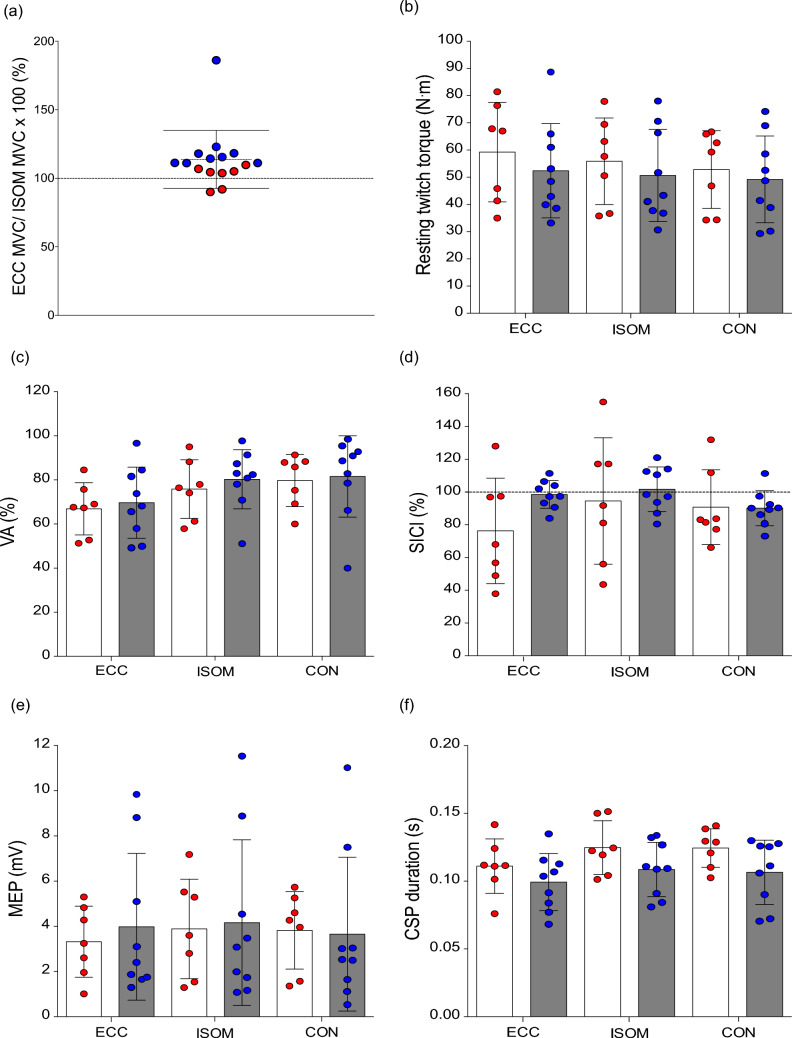
Ratio between maximal voluntary contraction (MVC) eccentric torque (ECC MVC) and isometric torque (ISOM MVC) (**a**), resting twitch torque (**b**), voluntary activation (VA) (**c**), short-interval intracortical inhibition (SICI; paired-pulse/single pulse MEP) (**d**), motor-evoked potential peak-to-peak amplitudes (MEP) (**e**), and corticospinal silent period (CSP) duration (**f**) during eccentric (ECC), isometric (ISOM) and concentric (CON) muscle contractions of the knee extensors at 30% MVCs. White bars indicate mean ± SD values of the individuals who had low ECC relative to ISOM MVC torque (Group A: *n* = 7). Grey bars indicate mean ± SD values of the individuals who had high ECC relative to ISOM MVC torque (Group B: *n* = 9). Data of group A individuals are shown in red circles, and data of Group B individuals are shown in blue circles

The distribution of the individuals based on ECC/ISOM MVC torque is shown in Fig. 6a. Two participants showed lower MVC torque in eccentric than isometric trials, but others (n = 14) showed greater MVC torque in eccentric compared to isometric contractions. No group x contraction type interaction nor group effect was evident for resting twitch torque (Fig. 6b), VA (Fig. 6c), SICI (Fig. 6d), MEP (Fig. 6e) and CSP duration at 30% MVC (Fig. 6f) [all *p* > 0.05].

## Discussion

The present study explored neurophysiological mechanisms that might explain the lower voluntary drive despite the greater force production during knee extensor eccentric than isometric and concentric contractions. We hypothesized that eccentric contractions would be accompanied by more intracortical inhibition. As expected, knee extensor MVC torque was greater in eccentric contractions, while VA was lower when compared with the other contractions tested (Figs. 2a and 3c). Resting twitch torque was also greater during passive lengthening than shortening and isometric (rest) conditions, indicating that the muscle fibers produced more tension during eccentric contractions (Fig. 3a). With cortical stimulation, MEP amplitudes were smaller and the CSP was shorter in duration for eccentric than other contractions (Figs. 5c and d). However, in contrast to our hypothesis, SICI did not differ between contraction types (Fig. 5f). Thus, it seems that the reduced VA during maximal eccentric contractions of the knee extensors is partly explained by the reduced MEP amplitude. In contrast, the decrease in CSP may indicate extra muscle spindle excitation of the motoneurons during eccentric contractions. In addition, individuals with lower ECC/ISOM MVC torque did not display different levels of neural inhibition, corticospinal excitability, voluntary drive or twitch responses when compared to those with greater ECC/ISOM MVC torque (Fig. 6).

MVC torque during isokinetic eccentric contractions of the knee extensors at 30°/s was on average 14% greater than that of isometric contractions and 33% greater than concentric contractions (Figs. 1 and 2). It has been suggested that during eccentric contractions, the myosin heads are able to re-bind to the active sites of actin filaments very quickly, which may explain why muscles are able to develop high forces with lower energy cost (Lombardi and Piazzesi 1990; Franchi et al. 2017). In addition, titin may also play a role in the greater force produced during eccentric contractions (Franchi et al. 2017; Nishikawa et al. 2012). The difference between eccentric and concentric MVC torque was in line with the study by Beltman et al. (2004) who reported that eccentric MVC torque of the knee extensors was 26% greater than concentric MVC torque, but in contrast to the present study, they showed no difference between eccentric (270 ± 55 Nm) and isometric MVC torque (252 ± 47 Nm).

Some studies have reported that greater force is produced during eccentric contractions with lower EMG activity (Duchateau and Baudry 2014; Duchateau and Enoka 2016; Beltman et al. 2004). In the present study, EMG activity (RMS) was not different between eccentric, isometric and concentric contractions. However, EMG relative to MVC torque (EMG/torque) was 16% smaller during eccentric than isometric, and 43% smaller during eccentric than concentric contractions (Fig. 2c). This was comparable to the study by Kellis and Baltzopoulos (1998) who reported that the integrated EMG activity of vastus lateralis normalized to MVC torque was 10–52% lower during eccentric than concentric contractions of the knee extensors at velocities ranging from 30°/s to 150°/s. During 30% MVC contractions, we also found that EMG/torque was 27% smaller during eccentric than isometric, and 63% smaller during eccentric than concentric contractions (Fig. 2d). Thus, it is likely that greater maximal and submaximal forces were produced with similar muscle fiber activity of the vastus lateralis during eccentric than isometric and concentric contractions.

Interestingly, individuals who produced relatively greater MVC torque in eccentric than isometric contractions did not display greater muscle activation than individuals who produced relatively smaller MVC torque in eccentric contractions (Fig. 6). The comparison between the two groups was based on a sub-analysis of the individuals who had low eccentric relative to isometric MVC torque (n = 7) and who had high eccentric relative to isometric MVC torque (n = 9). It should be noted that the sample size for this analysis would have been underpowered, since no sample size estimation was performed for this purpose initially. Thus, it would be interesting to further investigate whether differences in the tested variables exist between small and large ECC/ISOM MVC torque individuals by increasing the sample size in future studies. However, at least in the present study, it does not appear that larger ECC/ISOM MVC torque levels were underpinned by greater muscle activation. This was indicated by similar VA for the two groups, as well as similar EMG activity at 30% and 100% MVC for eccentric as for concentric and isometric contractions (Fig. 6c). These results seem to suggest that individuals who have higher eccentric torque relative to isometric torque have a greater contribution from muscle–tendon visco-elastic properties enabling a greater muscle force production and transmission rather than neurophysiological differences. Since no measurements relating to the muscle-joint complex were explored in the present study, it was not known how peripheral factors at the muscle, tendon and joint levels affected the force production capability in eccentric contractions among participants. Tecchio et al. (2024) have reported that eccentric exercises result in shortening–stretch contractions at the fascicle level, and the amount of fascicle shortening and stretch depends on the preactivation during the exercise. In the present study, each eccentric contraction was preceded by ~ 1 s maximal voluntary isometric contraction at 40° knee flexion. It is possible that differences in the level of the preactivation and fascicle behavior also affected the intra- and inter-individual force levels. Further studies are warranted to elucidate fascicle and muscle–tendon behaviors in different contraction types in different muscles, in relation to force output.

Although absolute levels of EMG activity were similar across contraction types, VA measured with twitch interpolation was lowest during eccentric MVCs. As shown in Fig. 3, resting twitch torque was 7% greater in passive lengthening after the eccentric MVCs compared to during passive shortening after concentric MVCs. However, superimposed twitch torque was 34–49% greater in eccentric than isometric and concentric MVCs, so that VA was 16–19% lower. This lower VA occurred despite participants commencing the eccentric MVCs after ~ 1 s of maximal isometric contraction at 40° knee extension. Previous studies have also shown that VA is lower during eccentric than isometric and/or concentric contractions (Beltman et al. 2004; Westing et al. 1990; Babault et al. 2001; Gravel et al. 1987) with knee extensor VA showing similar deficits to those in the current study (Beltman et al. 2004; Westing et al. 1990). For instance, Beltman et al. (2004) reported that VA was 14–15% lower during eccentric than isometric and concentric MVC of the knee extensors.

Potentially, cortical and/or spinal mechanisms could contribute to reduced VA during eccentric contractions. Previous studies have reported that responses to cortical stimulation, such as MEPs (Duclay et al. 2011; Gruber et al. 2009; Škarabot et al. 2018), CSP duration (Doguet et al. 2017; Duclay et al. 2011, 2014), and intracortical inhibition (i.e., SICI) (Opie and Semmler 2016) are modulated with contraction type and could be associated with the lower voluntary drive exhibited during eccentric than isometric and/or concentric contractions. Consistent with previous studies of the elbow flexors (Abbruzzese et al. 1994; Gruber et al. 2009), the current study found that vastus lateralis MEP amplitude was lower during eccentric than concentric and isometric MVCs. Furthermore, Doguet et al. (2017) reported that MEPs in vastus lateralis were lower during eccentric than isometric and concentric MVCs at 75° of knee flexion, but did not differ between muscle contractions at 100° of knee flexion.

In contrast, a study of the plantar flexors showed that MEP amplitude measured during submaximal (30% MVC) contractions did not differ between contraction types (Duclay et al. 2014). MEP amplitude depends on excitability at both cortical and spinal levels. At a cortical level, decreased excitability or increased inhibition could contribute to reduced MEP amplitude. The present study used paired pulse TMS to assess intracortical inhibition (SICI) and found no differences between contraction types. It should be noted that this assessment was performed during submaximal contractions where MEP amplitude was unaffected, thus it gives little insight into whether changes in MEP amplitude might be mediated by changes in SICI. Previously, differences in SICI have been reported, with less inhibition during weak eccentric than isometric contractions (EMG of ~ 6% MVC), but least inhibition during concentric contractions of the first dorsal interosseous muscle (Opie and Semmler 2016). However, in contrast to other limb muscles, MEPs in this hand muscle increase during lengthening contractions (Sekiguchi et al. 2007) so that changes in intracortical inhibition may also be muscle specific.

The CSP is a period of very low EMG activity following TMS and results from intracortical inhibition of descending drive combined with inhibitory mechanisms at a spinal level. Since spinal mechanisms contribute to the earlier part of the CSP (< 100 ms), changes in the duration of longer CSPs are thought to reflect changes in cortical inhibition (Butler et al. 2012; Škarabot et al. 2019; Ruas et al. 2022a). In the present study, CSP duration was often shorter than 100 ms and could be influenced by both spinal and cortical mechanisms. Nevertheless, the finding that the CSP was shorter during maximal and submaximal eccentric than isometric and concentric contractions supports previous findings from the plantar flexors (Duclay et al. 2011, 2014) and knee extensors (Doguet et al. 2017), with the latter measured at the same knee joint angle as that of the present study. The shortened CSP might be related to reduced MEP amplitude as changes in these two responses are often associated (Orth and Rothwell 2004). However, our finding of reduced CSP duration in submaximal eccentric contractions when MEPs were unchanged argues against this. One potential explanation for the shorter CSP duration during lengthening contractions is greater muscle spindle afferent firing that provides additional excitatory input to motoneurons during this period. Butler et al. (2012) found that for isokinetic contractions where muscle lengthening and shortening continued despite muscle relaxation during the CSP, low-level EMG during CSP was ~ 60–70% greater during eccentric than concentric MVC of the elbow flexors despite relaxation. They concluded that this was likely associated with spinal reflex facilitation caused by the firing of muscle spindles during muscle lengthening. Therefore, higher facilitatory input to the motoneurons during the CSP may result in them resuming firing more quickly to shorten the CSP during eccentric contractions.

Changes in excitability at a spinal level may also influence MEP amplitude, although this was not assessed in the present study. It has been reported that both H-reflexes and cervicomedullary motor evoked potentials (CMEPs), which are markers of motoneuron excitability, are reduced in lengthening contractions (Gruber et al. 2009; Duclay et al. 2011). Although inhibition at a spinal level seems counter to the argument above that spinal reflex facilitation from muscle spindle firing during muscle lengthening may shorten CSP duration, the motoneurons as well as the muscle spindles are in a different state during the CSP compared to during the ongoing contraction. During voluntary contractions, muscle spindles fire as a result of fusimotor drive combined with the superimposed changes in muscle length. While spindle firing may be greater during eccentric than concentric contractions, cortical control of presynaptic inhibition may reduce the level of afferent input to the motoneurons during eccentric contractions (Duchateau and Enoka 2016). By comparison, the CSP results in muscle relaxation and hence, muscle lengthening and spindle firing that is not anticipated by the nervous system and is, therefore, unlikely to be modulated prior to its facilitation of motoneurons.

Based upon our findings, the lower voluntary drive observed during eccentric contractions was not mediated by increased intracortical inhibition as shown by SICI or by CSP duration. However, the reduced MEP may reflect reduced excitability in the corticospinal pathway that may partly explain the poor voluntary activation during eccentric MVCs, despite the apparent decrease in inhibition indicated by the shortened CSP. As highlighted by Duchateau & Enoka (2016), it is possible that presynaptic and/or postsynaptic mechanisms at the motoneurons could modulate motor output during eccentric contractions. In turn, this could result in poorer VA compared to other muscle contraction types. However, evidence for specific spinal mechanisms is limited. Thus, mechanisms underpinning the greater reduction in VA during knee extensor eccentric than concentric and isometric contractions require further investigation. Furthermore, the neuromuscular and neurophysiological mechanisms that explain the greater force production in eccentric than other contractions warrant more studies.

There were several limitations in the present study. No familiarization session was included in the present study, since a previous study (Hibbert et al. 2020) reported no benefit of inserting one or two practice days prior to testing MVC torque of the knee extensors in young healthy participants, and additional testing days could even negatively impact subsequent strength measurements. In the present study, participants had practice trials prior to performing MVCs for each contraction mode, which were intended to optimize their maximal performance during the force measures. Although most participants of the present study had previous experience undertaking electrical nerve stimulations during muscle contractions, it is still possible that the lack of a proper familiarization session reflected on the relatively low voluntary activation levels that were observed in some individuals of the sample (Shield and Zhou 2004). It is also possible that the relatively low levels of VA were due to the compliance or distensibility of the isokinetic dynamometer used in the present study, which may affect the dissipation of force during knee extension MVC torque assessments when compared to custom-built (rigid) dynamometers (Maffiuletti et al. 2016).

We did not assess the test–retest reliability of the TMS measurements in the present study. However, we previously reported that TMS measurements (MEP and SICI) of the vastus lateralis assessed during submaximal isometric muscle contractions and a lower number of stimuli (five trials per intensity) were moderately reliable and did not differ across days (Ruas et al. 2020), in which the same investigators used similar participants to those of the present study. Although only isometric contractions were assessed in the study (Ruas et al. 2020), it demonstrated that TMS measurements would provide reasonable consistency within days or trials in this muscle. Thus, we assumed that this would also be the case for the dynamic contractions used in the present study, and that the measurements would allow us to compare the isometric, concentric and eccentric contractions that were performed in the same day. Nonetheless, it is important to examine the test–retest reliability of the TMS measurements during dynamic contractions, since they are likely different from static ones. It is also important to note that although there were no significant differences in the angles of concentric (84 ± 9°) and eccentric (83 ± 9°) MVC torques, these angles were significantly greater than the angle used for isometric MVC torque (75°). In addition, the peak torque angles in eccentric and concentric contractions were not the same as the 75° angle used for TMS and electrical stimulations. The 75° angle was chosen because a previous study found that MEP and CSP were different when assessed in a mid-angle (75° of knee flexion) than at a longer angle (100° of knee flexion) during concentric, eccentric and isometric contractions (Doguet et al. 2017). However, it is possible that these responses would be different if the stimuli were delivered at the exact angle of peak torque for each contraction type, which needs to be further examined.

The present study included male and female participants, but no comparison between sexes was made due to the small sample size that might not be adequate to perform sex comparisons. As shown in a supplementary table (Supplementary Table 1), when “sex” was included as an additional factor in our ANOVA analyses, no significant effects of sex were found for any variable (p ≥ 0.07). However, as only four females were included in the sample of the study, the supplementary analysis results require extreme caution. Thus, it is important to further examine whether specific sex differences exist for neuromuscular and neurophysiological characteristics under different muscle contractions. Lastly, a previous study reported that there were small but significant differences in the medial–lateral locations of TMS hotspots across cortical representations of quadriceps muscles (Davies 2020). Since we were not certain if the location and intensity of the stimuli were also optimal for the other quadriceps muscles we restricted TMS (and EMG) assessment and analyses of the study to the vastus lateralis muscle only, which may limit conclusions regarding the influence of these variables to the entire quadriceps muscle group. In addition, the lack of measurements from the other quadriceps muscles did not allow to understand the contribution of the synergistic muscles to the overall torque output during different contraction types. Therefore, the comparison of neuromuscular differences across muscle contractions in other specific quadriceps muscles may be of interest in future studies.

In conclusion, knee extensor MVC torque production was greater for eccentric than isometric and concentric contractions, but occurred despite reduced VA and similar muscle activity. Our findings of greater MVC and twitch torque, and greater torque for the same level of muscle activity during eccentric contractions are consistent with differences in muscle-level mechanical factors. On the contrary, poor VA was partly aligned with reduced corticospinal excitability, but was not explained by greater intracortical inhibition, which indicates that unexplored inhibitory mechanisms at level of the motoneurons are likely to contribute to the modulation of motor output during eccentric contractions of the quadriceps.

## Supplementary information

Below is the link to the electronic supplementary material.

Supplementary file1 (DOCX 20 kb)

## Data Availability

All data generated or analyzed during this study are included in the article.

## Abbreviations

CON: Concentric
CSP: Corticospinal silent period
ECC: Eccentric
EMG: Electromyographic
ISOM: Isometric
MEP: Motor-evoked potential
MVC: Maximal voluntary contraction
M_MAX_: Maximum (peak-to-peak amplitude) M-wave
M-wave: Compound muscle action potentials
SICI: Short-interval intracortical inhibition
TMS: Transcranial magnetic stimulation
VA: Voluntary activation
