# Supplementary material for: Neuromuscular characteristics of eccentric, concentric and isometric contractions of the knee extensors
Source: Eur J Appl Physiol. 2024 Oct 5;125(3):671–86. doi: 10.1007/s00421-024-05626-9 (PMC11889023; doi:10.1007/s00421-024-05626-9)
Supplement: Supplementary file 1 — Supplementary file1 (DOCX 20 kb) [file 421_2024_5626_MOESM1_ESM.docx]

**Supplementary Table 1.** Dependent variables for eccentric (ECC), isometric (ISOM) and concentric (CON) contractions of male (n = 12) and female (n = 4) participants of the study. No significant contraction*sex interactions were found for any variable (p > 0.05).

|  | ECC | | | ISOM | | CON | |  |
| --- | --- | --- | --- | --- | --- | --- | --- | --- |
|  |  | **Male** | **Female** | **Male** | **Female** | **Male** | **Female** | ***P*** |
|  |  |  |  |  |  |  |  |  |
| MVC Torque | *Mean* | 329.0 | 235.9 | 302.3 | 182.8 | 264.9 | 156.6 | 0.48 |
| (N^.^m) | *± SD* | ± 90.8 | ± 65.9 | ± 74.1 | ± 30.4 | ± 68.4 | ± 61.4 |  |
|  | *Range* | (208.1 - 535.7) | (162.9 - 311.4) | (74.16 - 481.8) | (156.0 - 225.4) | (186.6 - 431.1) | (156.0 - 225.4) |  |
| 30% of MVC Torque | *Mean* | 115.7 | 73.9 | 97.3 | 61.7 | 77.5 | 57.0 | 0.13 |
| (N^.^m) | *± SD* | ± 32.8 | ± 17.9 | ± 25.5 | ± 11.7 | ± 17.5 | ± 23.2 |  |
|  | *Range* | (67.6 - 184.5) | (54.3 - 97.1) | (74.9 - 163.2) | (47.3 - 76.0) | (55.3 - 108.8) | (43.0 - 91.5) |  |
| EMG/torque at 100% MVC | *Mean* | 1.9 | 1.6 | 2.1 | 1.8 | 2.6 | 2.5 | 0.82 |
| (μV/N^.^m) | *± SD* | ± 1.2 | ± 0.6 | ± 1.3 | ± 0.4 | ± 1.7 | ± 1.3 |  |
|  | *Range* | (0.7 - 4.6) | (0.9 - 2.3) | (0.8 - 5.4) | (1.1 - 2.1) | (0.9 - 6.4) | (1.6 - 4.3) |  |
| EMG/torque at 30% MVC | *Mean* | 3.1 | 2.7 | 3.8 | 4.2 | 4.8 | 4.4 | 0.34 |
| (μV/N^.^m) | *± SD* | ± 2.5 | ± 3.1 | ± 3.0 | ± 4.4 | ± 3.6 | ± 4.7 |  |
|  | *Range* | (0.6 - 7.2) | (0.9 - 7.3) | (0.8 - 7.9) | (1.5 - 10.8) | (1.1 - 9.9) | (1.7 - 11.4) |  |
| Resting twitch torque | *Mean* | 60.4 | 38.9 | 58.9 | 35.2 | 56.4 | 33.2 | 0.85 |
| (N.m) | *± SD* | ± 17.5 | ± 4.3 | ± 14.9 | ± 3.2 | ± 12.6 | ± 4.4 |  |
|  | *Range* | (34.9 - 88.7) | (33.2 -42.9) | (36.7 - 78.1) | (30.6 - 37.7) | (34.3 - 74.2) | (29.3 - 38.8) |  |
| Superimposed twitch torque | *Mean* | 18.9 | 13.9 | 12.8 | 8.8 | 11.1 | 6.5 | 0.88 |
| (N.m) | *± SD* | ± 12.8 | ± 6.4 | ± 11.1 | ± 4.9 | ± 13.1 | ± 3.4 |  |
|  | *Range* | (2.0 - 45.2) | (5.9 - 20.1) | (1.6 - 38.0) | (3.2 - 15.1) | (0.9 - 44.5) | (1.8 - 9.4) |  |
| VA | *Mean* | 69.9 | 64.8 | 79.9 | 74.5 | 82.5 | 78.8 | 0.92 |
| (%) | *± SD* | ± 15.0 | ± 14.3 | ± 13.8 | ±13.9 | ± 17.1 | ± 12.2 |  |
|  | *Range* | (49.1 - 96.7) | (51.3 - 84.5) | (51.1 - 97.7) | (57.9 - 91.4) | (40.0 - 98.5) | (66.2 - 95.5) |  |
| MEP at 100% MVC | *Mean* | 3.7 | 2.7 | 4.4 | 3.7 | 4.1 | 3.6 | 0.64 |
| (mV) | *± SD* | ± 1.8 | ± 1.2 | ± 2.5 | ± 2.1 | ± 2.1 | ± 2.2 |  |
|  | *Range* | (1.1 - 6.3) | (1.7 - 4.4) | (1.0 - 8.2) | (2.3 - 6.9) | (1.0 - 7.8) | (2.0 - 6.8) |  |
| MEP at 30% MVC | *Mean* | 3.9 | 2.6 | 4.3 | 2.9 | 3.9 | 2.9 | 0.89 |
| (mV) | *± SD* | ± 2.9 | ± 1.2 | ± 3.4 | ± 1.9 | ± 3.2 | ± 1.1 |  |
|  | *Range* | (1.0 - 9.8) | (1.7 - 4.3) | (1.1 - 22.5) | (1.2 - 5.5) | (0.5 - 11.0) | (1.6 - 4.3) |  |
| CSP duration at 100% MVC | *Mean* | 0.10 | 0.08 | 0.11 | 0.11 | 0.12 | 0.09 | 0.07 |
| (s) | *± SD* | ± 0.02 | ± 0.03 | ± 0.02 | ± 0.02 | ± 0.03 | ± 0.02 |  |
|  | *Range* | (0.06 - 0.15) | (0.06 - 0.20) | (0.08 - 0.14) | (0.08 - 0.13) | (0.08 - 0.17) | (0.06 - 0.11) |  |
| CSP duration at 30% MVC | *Mean* | 0.11 | 0.09 | 0.12 | 0.11 | 0.12 | 0.10 | 0.65 |
| (s) | *± SD* | ± 0.02 | ± 0.02 | ± 0.2 | ± 0.03 | ± 0.02 | ± 0.03 |  |
|  | *Range* | (0.08 - 0.14) | (0.07 - 0.11) | (0.08 - 0.15) | (0.08 - 0.15) | (0.07 - 0.14) | (0.07 - 0.13) |  |
| M_MAX_ | *Mean* | 9.7 | 7.8 | 10.5 | 9.0 | 9.4 | 8.2 | 0.82 |
| (mV) | *± SD* | ± 5.1 | ± 7.0 | ± 5.4 | ± 6.6 | ± 5.6 | ± 6.9 |  |
|  | *Range* | (3.1 - 19.3) | 2.8 - 18.1) | (3.1 - 20.1) | (3.1 - 18.4) | (3.3 - 19.5) | 3.3 - 19.5) |  |
| SICI | *Mean* | 0.8 | 0.8 | 0.9 | 0.8 | 0.8 | 0.8 | 0.97 |
| (%) | *± SD* | ± 0.2 | ± 0.3 | ± 0.1 | ± 0.1 | ± 0.1 | ± 0.1 |  |
|  | *Range* | (0.2 - 1.3) | (0.4 - 1.0) | (0.7 - 1.12) | (0.7 - 1.1) | (0.6 - 0.9) | (0.7 - 0.9) |  |

Abbreviations: Maximal voluntary contraction (MVC), vastus lateralis muscle electromyographic (EMG) activity normalized to torque (EMG/torque), voluntary activation (VA), motor evoked potential (MEP) corticospinal silent period (CSP) duration M_MAX_, short interval intracortical inhibition (SICI).
